# Corynebacterium jeikeium as an Unusual Cause of Keratitis: A Case Report From a Tertiary Care Hospital in Chhattisgarh, India

**DOI:** 10.7759/cureus.20164

**Published:** 2021-12-04

**Authors:** Vijaya Sahu, Madhu Mallika Pathak, Padma Das, Ashik Ravi

**Affiliations:** 1 Ophthalmology, All India Institute of Medical Sciences, Raipur, IND; 2 Microbiology, All India Institute of Medical Sciences, Raipur, IND

**Keywords:** corynebacterium jeikeium, trauma, vancomycin, corneal ulcer, immunocompetent, non-pathogenic

## Abstract

Bacterial keratitis is a serious, potentially sight-threatening complication in neglected cases of corneal trauma. Few bacteria and fungi are implicated in the pathogenesis of bacterial keratitis after trauma; however, keratitis by an opportunistic organism is rare. We report here a case of keratitis caused by Corynebacterium jeikeium (C. jeikeium) after accidental trauma with rice grains. The patient presented to us with chronic keratitis, which responded to 5% fortified vancomycin eye drops after microbiological investigation and drug sensitivity.

## Introduction

Bacterial keratitis may be sight-threatening and the severity of keratitis depends on the virulence of the organism and the condition of the cornea [1]. Sometimes normal flora too may cause ocular infection when microorganisms overpower host defenses but it is not so common.

Corynebacterium species is usually found in the skin and mucous membrane as a part of normal flora, and we consider it a contaminant when found in clinical samples [2]. They are usually non-pathogenic on the ophthalmic surface, even when isolated from the eyes with keratitis.

The possibility of human infection by these organisms is relatively rare, but in the last few years, the incidences of human infection have increased significantly. Some pathogenic species in human beings include - Corynebacterium amycolatum, Corynebacterium striatum, Corynebacterium jeikeium (C. jeikeium), Corynebacterium urealyticum, and Corynebacterium xerosis [3]. Endocarditis in patients with prosthetic valves has been documented by infection with C. jeikeium; however, involvement of the ocular surface is quite rare [4]. The main risk factors for C. jeikeium infection are prolonged hospitalization, use of multiple antibiotics over a period of time, and immunocompromised state [5].

We present a case of keratitis caused by C. jeikeium in an immunocompetent patient following trauma with rice grains. This is the first reported case of bacterial keratitis caused by C. jeikeium in an immunocompetent person.

## Case presentation

A 50-year-old lady reported to the ophthalmology outpatient department with complaints of diminution of vision in the right eye associated with pain, redness, photophobia, and watering for the past one month. She had a history of trauma with rice grains a few days before the initial presentation of ocular symptoms. She was treated with multiple topical eye drops like 0.5% moxifloxacin and 0.3% tobramycin, but the symptoms did not resolve, and she experienced worsening of symptoms. She had no past medical history like diabetic mellitus, hypertension, any surgical history, or any malignancy. The patient, when admitted under us, underwent routine viral markers investigations in which she was found negative for human immunodeficiency virus (HIV-1 and HIV-2) enzyme-linked immunosorbent assay (ELISA). The patient was not on oral steroids or any immunomodulators or any immunosuppressive drugs.

**Figure 1:**
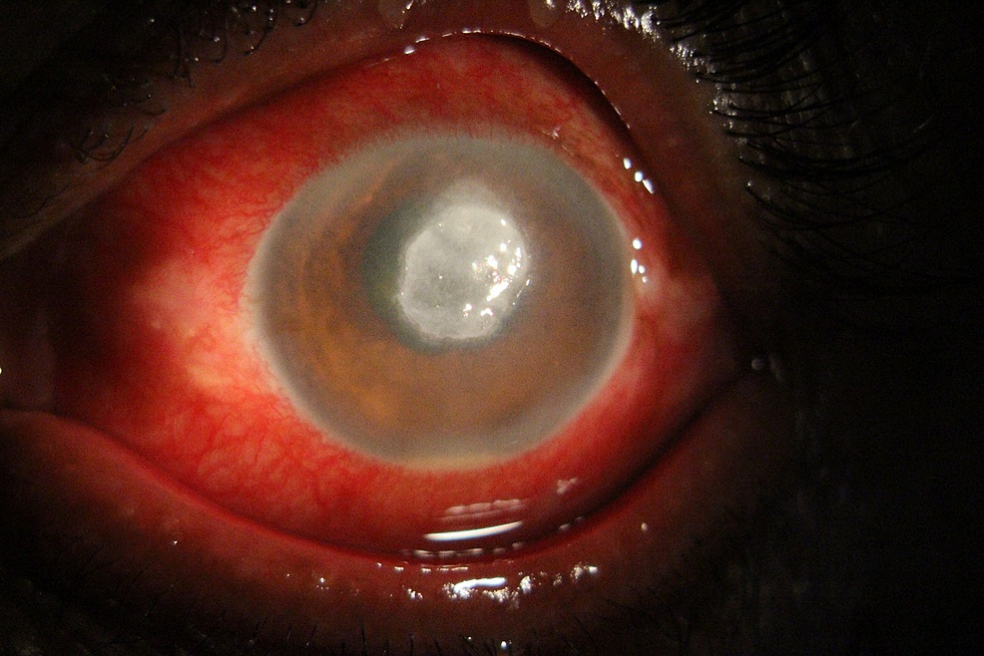
Photograph of the right eye with mixed type of congestion, central corneal ulcer with epithelial defect of size 5 x 4 mm, stromal infiltration of size 6 x 5 mm, with streak hypopyon.

Slit-lamp examination revealed mild lid edema in her right eye with mixed congestion, central corneal ulcer with epithelial defect of size 5 x 5 mm, stromal infiltration of size 6 x 5 mm, with streak hypopyon. There were no satellite lesions. The details of the lens and posterior segment could not be assessed due to the hazy view (Figure 1).

**Figure 2:**
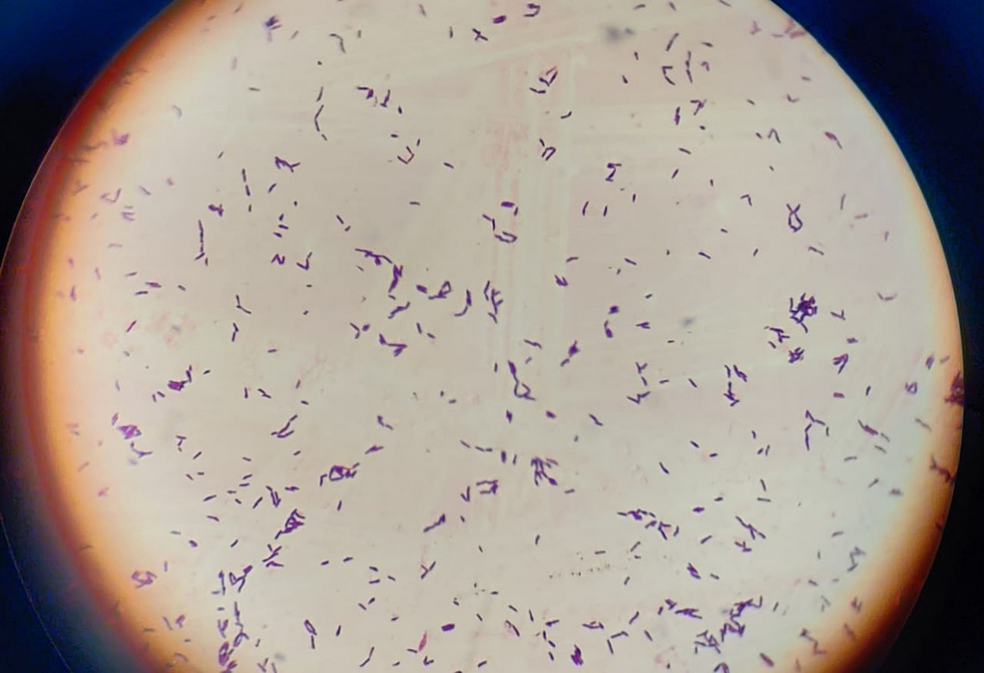
Photograph of a gram staining showing gram-positive rod-shaped bacteria with palisading.

The vision was counting fingers at two meters with accurate projection of rays in all four quadrants. Direct pupillary reaction assessed from the periphery was found to be absent due to previous use of atropine eye ointment. B scan was performed, which revealed an anechoic vitreous cavity. The microbiological investigation was done as per our routine protocol. The corneal scraping was obtained with a 15 number surgical blade under topical anesthesia. The specimen was sent for different microbiological processing like potassium hydroxide (KOH), gram stain, and inoculation on 5% sheep blood agar, 5% sheep blood chocolate agar, and Sabouraud dextrose agar (SDA). KOH mount was negative for fungal growth, and the gram stain of the specimen showed gram-positive rods and a moderate amount of pus cells (Figure 2).

**Figure 3:**
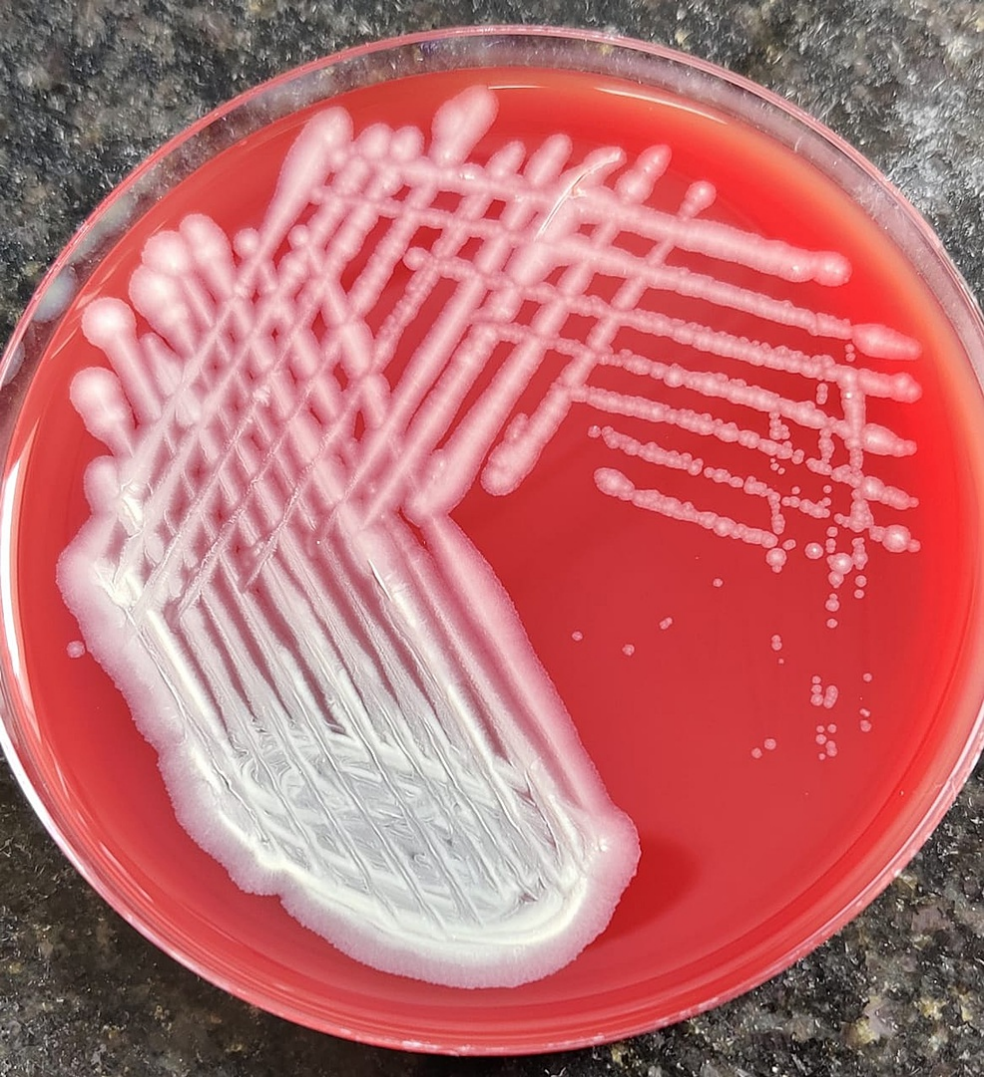
Photograph showing confluent and isolated Corynebacterium jeikeium colony on sheep blood agar.

We kept the patient on 5% fortified cefuroxime eye drops, 1.4% fortified tobramycin eye drops, and 1% atropine eye drops. But there were no signs of improvement after the start of treatment. After 72 hours of incubation in blood agar, the culture report showed the presence of C. jeikeium, which was only sensitive to vancomycin (Figure 3).

**Figure 4:**
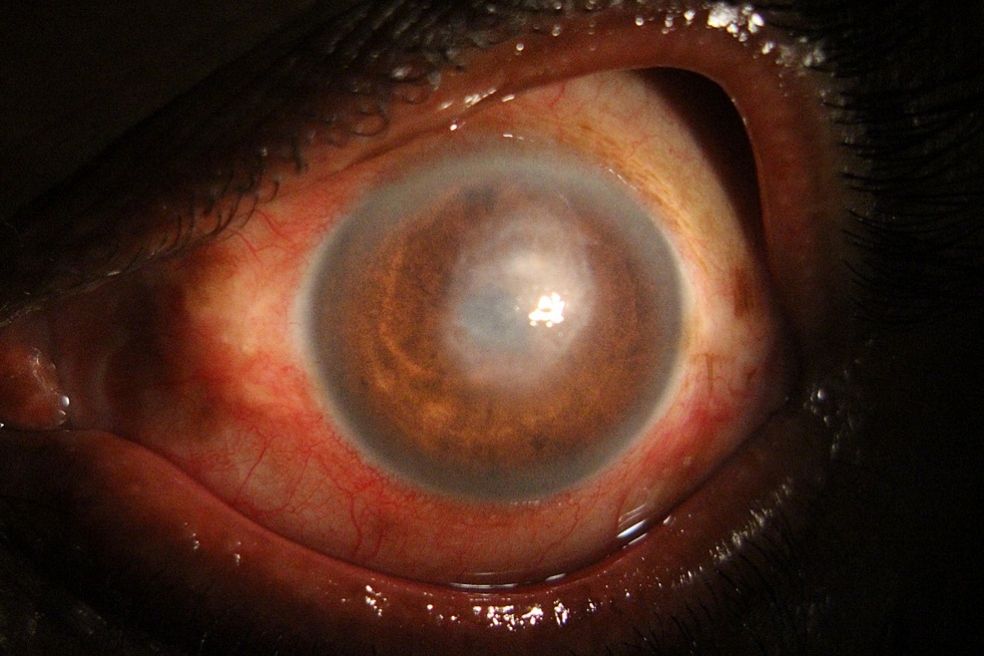
Healing of corneal ulcer with formation of macular corneal opacity.

After culture and sensitivity reports, we discontinued the fortified cefuroxime and fortified tobramycin eye drops and started an hourly topical application of 5% fortified vancomycin eye drops. The ulcer started healing after 48 hours of use of vancomycin eye drops. Streak hypopyon was also resolved. The visual acuity improved to counting fingers at four meters. After eight days, a reduction in the size of the infiltrate was noticed with symptomatic improvement, and the complete resolution was seen after three weeks, and vision improved to 6/36 with central macular opacity (Figure 4).

## Discussion

The genus Corynebacterium currently has more than 100 validated species which is highly diversified. It includes species with medical or biotechnological relevance. Collins and Cummins, in 1986, were the first to describe the principal features of the Corynebacterium genus. Coryneform may be associated with conjunctivitis, keratitis, and endophthalmitis [3]. However, they are also increasingly recognized as causing opportunistic disease under specific circumstances, like in immunocompromised patients or patients with prolonged hospitalization [5].

C. jeikeium is a recently described species that is an emerging nosocomial pathogen. It was initially identified by the Centre for Disease Control and Prevention (CDC) as group JK; later in 1988, it was designated as C. jeikeium [6]. C.jeikium is a gram-positive, aerobic bacillus, primarily present on the skin surface. Infections with C. jeikeium have been noted predominantly in immunosuppressed patients (mostly malignancies) or patients with medical devices (e.g., catheters). Other clinical manifestations may be pulmonary infiltrates, skin rashes, septic cutaneous emboli, and soft tissue infections [7]. But it has rarely been associated with serious ocular infections such as keratitis or endophthalmitis.

Recently, a case of endophthalmitis due to C. jeikeium in a 7-year-old female child following penetrating injury has been reported. However, the immune status of the said patient has not been mentioned [8]. Our patient was immunocompetent with no relevant past medical history or prolonged hospitalization; the only risk factor was trauma to the eye with rice grains, which is consistent with other studies suggesting trauma as a risk factor. Schaefer et al., in their research, found trauma as a risk factor in 20% of cases [9]. Similarly, Bourcier et al. have identified trauma as a risk factor in 15% of 300 patients [10]. The trauma probably rendered the cornea susceptible to infection by creating an appropriate environment to convert the commensal organism to pathogenic and helping in easy adherence of the organism. This could be the reason for the occurrence of keratitis in our patient [3]. Previous literature suggested that C. jeikeium is resistant to fluoroquinolones and is sensitive to vancomycin, which is also confirmed in this case [11].

## Conclusions

C. jeikeium is an unusual pathogenic species that may cause keratitis in an immunocompromised patient, mostly nosocomial infections. However, this case has demonstrated that C.jeikium can also cause keratitis in an immunocompetent patient. The predisposing factor for such infection is trauma. The management of such a case is critical as the organism is resistant to commonly used antibiotics. The meticulous microbiological evaluation and drug sensitivity may be vital for these types of non-healing keratitis.
